# An Efficient Algorithm Embedded in an Ultrasonic Visualization Technique for Damage Inspection Using the AE Sensor Excitation Method

**DOI:** 10.3390/s141120439

**Published:** 2014-10-29

**Authors:** Yaolu Liu, Riu Goda, Kiyoshi Samata, Atsushi Kanda, Ning Hu, Jianyu Zhang, Huiming Ning, Liangke Wu

**Affiliations:** 1 College of Aerospace Engineering, Chongqing University, Chongqing 400044, China; E-Mails: liuyl28@163.com (Y.L.); jyzhang@vip.126.com (J.Z.); ninghuiming@gmail.com (H.N.); zdliangke@163.com (L.W.); 2 Department of Mechanical Engineering, Chiba University, Chiba 263-8522, Japan; E-Mail: r.goda@chiba-u.jp; 3 JAL Engineering Co. Ltd., Haneda Airport Ota, Tokyo 144-0041, Japan; E-Mail: kiyoshi.samata@jal.com; 4 Japan Aerospace Exploration Agency, Osawa Mitaka, Tokyo 181-0015, Japan; E-Mail: kanda@chofu.jaxa.jp

**Keywords:** visualization technique, Lamb waves, kinetic energy, diagnostic image

## Abstract

To improve the reliability of a Lamb wave visualization technique and to obtain more information about structural damages (e.g., size and shape), we put forward a new signal processing algorithm to identify damage more clearly in an inspection region. Since the kinetic energy of material particles in a damaged area would suddenly change when ultrasonic waves encounter the damage, the new algorithm embedded in the wave visualization technique is aimed at monitoring the kinetic energy variations of all points in an inspection region to construct a damage diagnostic image. To validate the new algorithm, three kinds of surface damages on the center of aluminum plates, including two non-penetrative slits with different depths and a circular dent, were experimentally inspected. From the experimental results, it can be found that the new algorithm can remarkably enhance the quality of the diagnostic image, especially for some minor defects.

## 1. Introduction

The development of various powerful nondestructive inspection techniques to detect possible defects is crucial to improve the safety, reliability and operation life of various aged structures. To date, some techniques, e.g., X-ray inspection [1], infrared temperature measurement [2], thermography [3], eddy-current detection [4], Lamb wave tomography [5], ultrasonic C-scan [6], *etc.*, have been developed for this purpose. Among them, those methods based on ultrasonic waves [5–8] have been attracting increasing attention, and ultrasonic scanning [6] is probably one of the most commonly used techniques in practice. In this method, a probe scanning the surface of a structure generates bulk ultrasonic waves, which propagate along the specimen thickness direction. Internal structural defects can then be evaluated by analyzing the time-domain or frequency-domain signal characteristics of waves transmitted or reflected from the defects. However, its inspection region is relatively small and the inspection process is very time consuming. In addition, overlapping and interference of multiple reflected and diffracted waves are still technically challenging and inspection results may largely depend on the experience and skill of inspectors. It is quite possible to overlook or even misinterpret some types of defects.

To deal with these problems, some new damage monitoring or inspection techniques based on Lamb waves propagating over a long distance in structural span directions, have been recently developed [9–13]. Based on the laser scanning excitation method and Betti's reciprocal theorem, Takatsubo *et al.* [14–16] proposed a simple visualization technique to reproduce ultrasonic Lamb wave propagation for damage inspections. Compared with the conventional ultrasonic scanning methods using bulk waves, this technique can inspect a large area quickly. However, damages may be overlooked or misinterpreted when only monitoring the snapshots of the Lamb wave propagation in inspected structures at different time points. Moreover, detailed information about the damage, such as area and shape, cannot be provided.

To improve the reliability of the Lamb wave visualization technique, the present authors have proposed a new concept of wave energy flow (WEF) map to evaluate the shape and size of damaged areas [17]. The WEF map for damage inspection is basically a damage diagnostic image, which is constructed by employing a quantity, *i.e.*, an equivalent strain energy density. It is obtained by summing up the square of the time-series *strain data* of every grid point in an inspection region. This method was proven by various experimental results to be very effective [17]. In this work, we propose a new signal processing algorithm to further improve the quality of damage diagnostic images. It is known that the kinetic energies of material particles in a continuous medium change continuously and periodically when waves propagate through a perfect material. Also, it can be understood that the strain energy and kinetic energy of a particle interchange reciprocally during wave propagation (or wave energy propagation). However, the kinetic energies of the particles in a damaged area would change suddenly when the waves encounter the damage during the propagation process. Therefore, this new algorithm aims to obtain improved images of the damaged area by highlighting the kinetic energy difference of each grid point with its all neighboring points. The idea is basically similar to that of Moore neighbor tracing based on the concept of the Moore neighborhood of a pixel. This algorithm, which is very suitable for minor defects, was embedded in the Lamb wave visualization technique. Therefore, both wave propagation configuration at every time point and the information about the damaged region estimated by the new algorithm can be obtained simultaneously. This feature remarkably improves the inspection reliability, especially for some slight defects which cannot be identified by the Lamb wave visualization technique. To verify the effectiveness of the new algorithm, based on an AE sensor excitation method to generate Lamb waves, three aluminum plates containing three types of surface damage, *i.e.*, two non-penetrative slits with different depths and a circular dent, were employed in experiments.

This article is arranged as follows: Section 2 describes the signal data analysis method, including a detailed explanation of the new algorithm in Section 2.2. The experimental scheme is depicted in Section 3.1. The experimental investigation of various damages in aluminum plates are reported in Sections 3.2–3.4. Finally, some conclusions are drawn in Section 4.

## 2. Analytical Method

**Figure 1.**
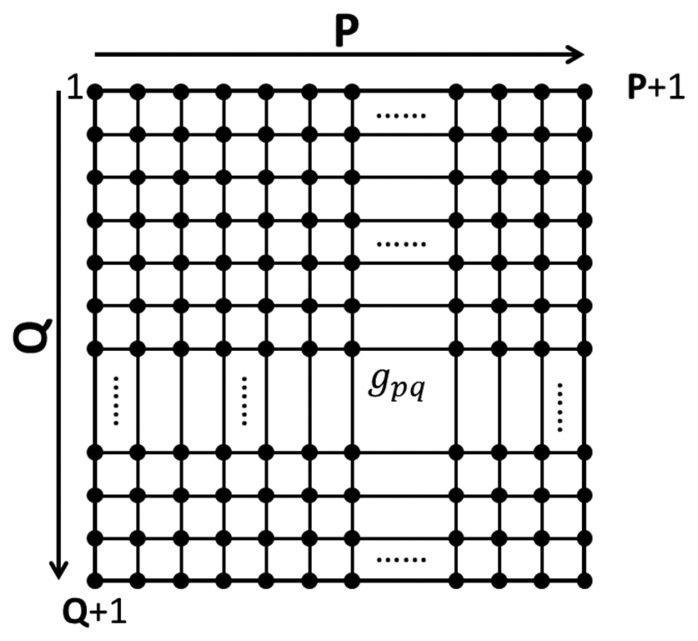
Grids of inspection region.

As shown in Figure 1, the inspection region is divided into P × Q grids. The grid points are marked as *g_pq_*, and 1 ≤ *p* ≤ P + 1, 1 ≤ *q* ≤ Q + 1. When ultrasonic Lamb waves propagate through the inspected region, the wave signals at every grid point are collected for imaging processing. The signal data analysis method involves two steps: the first step is to evaluate the kinetic energy or a kinetic energy equivalent quantity of every grid point; the second one is to extract the difference between the kinetic energy of each grid point and those of all its neighboring points.

### 2.1. Evaluation of Kinetic Energy

During a specified period, the total kinetic energy of each grid point or its equivalent quantity can be evaluated by summing up the amplitudes of the time-series wave signal collected at each grid point. Consider the case that the sampling time period for collecting wave signal is *T*[*s*], the time sampling interval is Δ*T*[*s*], and we can denote the signal value collected at the *i*-th time as *α_i_*. As explained later, since the numerical signal data obtained by experiment are usually very small values, those numerical values are multiplied by 10^7^ for easy handling. Therefore, the *i*-th amplified signal value marked as *β_i_* can be expressed as follows:

(1) $$\beta_{i}=\alpha_{i}\times 10^{7}$$

For the grid point *g_pq_*, the total kinetic energy marked as *γ_pq_* can be estimated by summing up the time-series signal data as follows:

(2) $$\begin{array}{cc} \gamma_{pq}=\overset{n}{\underset{i=1}{\text{∑}}}\left|\beta_{i}\right|, & n=\frac{T}{\Delta T} \end{array}$$

By repeating the above computation to obtain the kinetic energies of all grid points, a kinetic energy distribution image can be constructed for damage diagnosis. Compared to that of monitoring the snapshots of ultrasonic wave propagation to identify damage [14–16], the present method only needs the cumulative operation of signal data in a certain period of time, which is easier for inspectors although the snapshots of ultrasonic wave propagation also can be reproduced. Basically, in our previous work [17], the WEF map for damage inspection was constructed using Equation (2) although the square operation was used instead of the absolute operation in Equation (2). Moreover, the sensor signal was obtained from a lead zirconate titanate (PZT) sensor for strain measurement [17].

### 2.2. Comparison of Kinetic Energy

**Figure 2.**
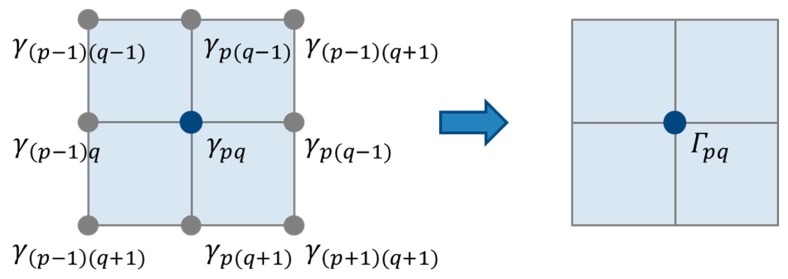
Schematic view of the new algorithm.

To further improve the reliability of the Lamb wave propagation visualization technique [14–16] and avoid overlooking any damage, unlike [17], a new signal processing algorithm embedded in the visualization technique is proposed to make the image of the damaged area become more clear. This algorithm using the sensor data of “kinetic energy” or its equivalent quantity is similar to the Moore neighbor tracing method using the concept of the Moore neighborhood of a pixel. It is known that the propagation of elastic waves is dependent on the kinetic motion of mass particles, which can be transferred smoothly from one particle to the next one in a smooth and continuous medium. However, when a defect exists in the medium resulting in the discontinuity, the kinetic energy transmission of the particles in or near the damage area would be hindered, leading to a sudden change in the kinetic energy for those particles. Therefore, by measuring the difference between the kinetic energy of a particle and its neighboring points, *i.e.*, the Moore neighborhood, the image quality of damage area can be enhanced compared to that obtained only using Equation (2) in Section 2.1. As shown in Figure 2, taking an arbitrary grid point g_pq_ inside an inspected region as an example, there are eight neighboring points around the grid point *g_pq_*, which are *g*_(_*_p_*_−1) (_*_q_*_−1)_, *g*_(_*_p_*_−1)_*_q_*, *g*_(_*_p_*_−1) (_*_q_*_+1)_, *g_p_*_(_*_q_*_−1)_, *g_p_*_(_*_q_*_+1)_, *g*_(_*_p_*_+1) (_*_q_*_−1)_, *g*_(_*_p_*_+1)_*_q_*, *g*_(_*_p_*_+1) (_*_q_*_+1)_, respectively. The kinetic energies of those grid points are expressed as *γ_ij_*, where *i* = *p* − 1, … ,*p* + 1 and *j* = *q* − 1, … ,*q* + 1. To quantify the difference of the kinetic energy of a particle and its neighboring points, a measuring value *Γ_pq_* for the grid point *g_pq_* was defined by Equation (3) and illustrated in Figure 2.

For a grid point at the edge of the inspection region, although the number of its neighboring points is less than eight, its measurement value *Γ_pq_* can still be calculated using Equation (3) by only considering its current neighboring points.

(3) $$\Gamma_{pq}=\left|\overset{p+1}{\underset{i=p-1}{\text{∑}}}\overset{q+1}{\underset{j=q-1}{\text{∑}}}\left(\gamma_{ij}-\gamma_{pq}\right)\right|$$

## 3. Experiment and Results

### 3.1. Experimental Scheme

**Figure 3.**
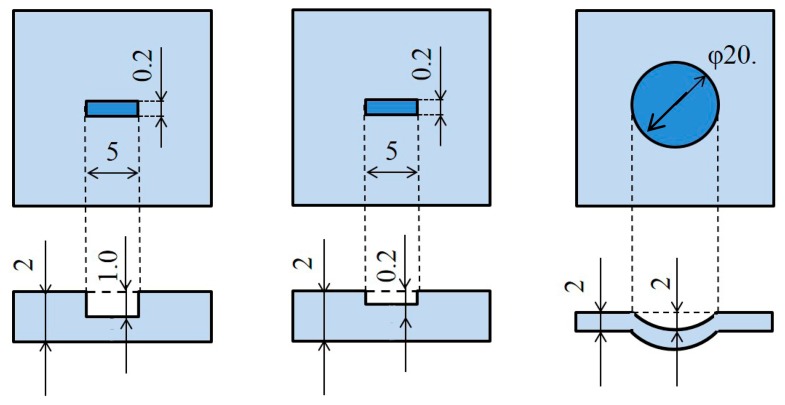
Three kinds of surface damages.

**Figure 4.**
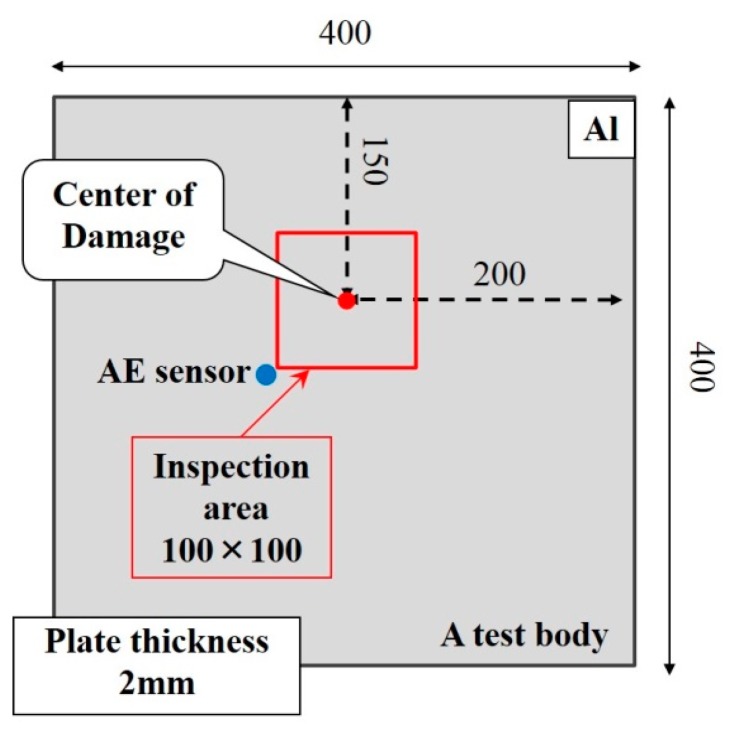
Schematic of an aluminum plate and AE sensor in the experiments (unit: mm).

To confirm the validity of the new algorithm, three kinds of surface damage on the center of aluminum plates, including two non-penetrative slits with different depths and a circular dent, were experimentally inspected. The damages are schematically illustrated in Figure 3. The depths of the two non-penetrating slits were 1 mm and 0.2 mm, respectively, and the diameter and depth of the circular dent were 20 mm and 2 mm. The sizes of the square aluminum plates used in experiments were 400 mm (length) × 400 mm (width) × 2 mm (thickness) and the dimensions of inspection region with damage inside were 100 mm × 100 mm. The inspection region was divided into 50 × 50 square grids with an interval of 2 mm, leading to 51 × 51 grid points. The wave function was generated by a function generator (Multifunction Generator WF1974, NF Co., Yokohama, Kanagawa, Japan), which was amplified by an amplifier (BA4825, NF Co.). Then the amplified function was used to drive an AE sensor (R6 (resonance frequency: 60.0 kHz), Physical Acoustics Co., Princeton, NJ, USA). As shown in Figure 4, the AE sensor fixed outside the inspection region was used as *actuator* to excite Lamb waves in the plates. The excitation frequency of AE sensor was 93.5 kHz. Moreover, a velocity decoder (PSV500, Polytec Inc., Irvine, CA, USA) was used as sensor to scan every grid point in the inspection region to collect ultrasonic wave signals. Since the output of the velocity decoder is the velocity *v* of a particle in the plate at different times, the displacement *S* of the particle at different times are obtained by performing numerical integration which can be expressed in the following equation:

(4) $$S(t)=\int_{t_{1}}^{t_{2}}\text{vdt}$$

Before calculation, we firstly applied high-pass filter processing to the original output data in order to remove the DC offset component, and then implemented a zero phased FIR filter to remove aliasing noise at a sampling time. The precision of the displacement obtained by numerical integration depends on integration time interval and the precision of velocity measurement. For the integration time interval, taking a waveform being slightly lower than 100 kHz as an example, by setting a sampling frequency as 2.56 MHz, the integration time interval is short enough when calculating the displacement. For the precision of velocity measurement, the velocity decoder was sufficiently calibrated before its practical use, and the calibration error of the voltage conversion to velocity is about 0.2%, which is much less than the error tolerance of 2.0% required in the present experiments.

By measuring the vibration velocity at each gird point and integrating it, the amplitude of the vibration at the point can be estimated. Therefore, the waveform data contain the transverse displacements of the all points in the inspected region at different moments, which were used for image processing by using Equations (2) and (3). Note that, when using the vibration amplitude, the quantity evaluated in Equation (2) can be considered as a scale to measure kinetic energy, or elastic potential energy of particles since basically, these two energies interchange reciprocally during wave propagation. In the experiment, the sampling time for collecting wave signals was 400 μs, and the sampling frequency was 2560.0 kHz.

### 3.2. Waveform Comparison

**Figure 5.**
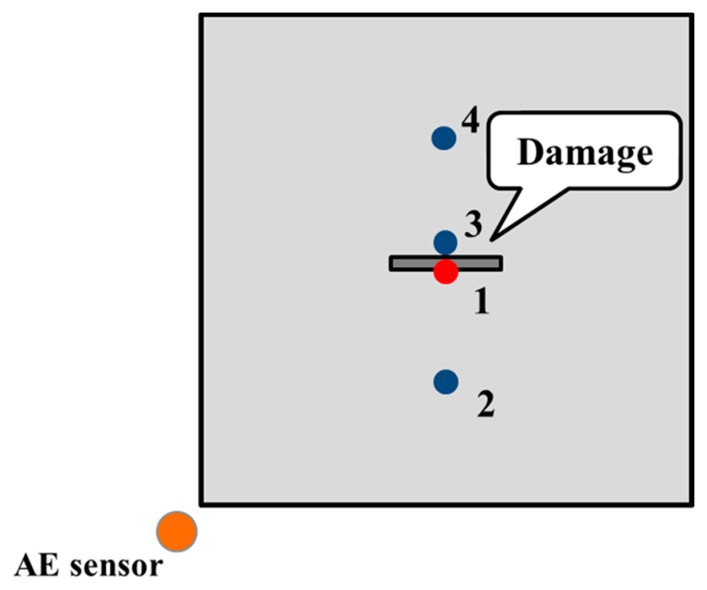
Positions of four inspection points.

**Figure 6.**
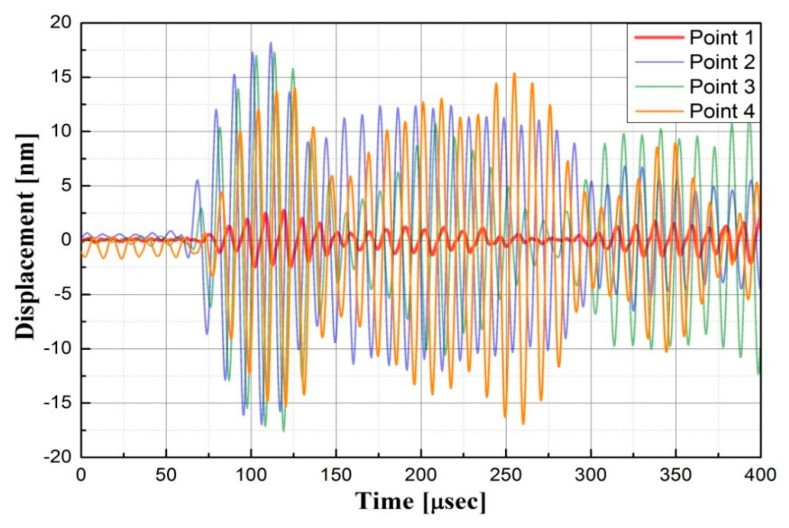
Waveforms collected at Points 1–4.

To construct the diagnostic damage image of the inspection region from wave signals, the waveform characteristics were investigated first to study the influence of damage. Consider the case of an aluminum plate with a 1 mm depth non-penetrative slit; we measured the waveform data of four points as shown in Figure 5, where Point 1 was located at the front edge of the slit, Point 2 was at the front of the slit and far away from it, Point 3 was behind the slit and very close to it, and Point 4 was also behind the slit, but far away from the slit. Figure 6 demonstrates the waveforms collected at the four points. It can be seen that the wave amplitude of Point 1 is much smaller than that of the Point 2, which might be caused by the reflections from the slit. Interestingly, the wave amplitudes of Point 3 and Point 4 are not so small like that of Point 1. Therefore, the “shadow” effect seems to be not so obvious in the experiment, probably due to the present extremely narrow and shallow slit. By virtue of this characteristic of wave amplitude change caused by a defect, it is expected that the image quality of the damage area can be improved when using the Moore neighbor tracing method as described in Equation (3).

### 3.3. Experimental Results for a Non-Penetrative Slit (1 mm Depth)

**Figure 7.**
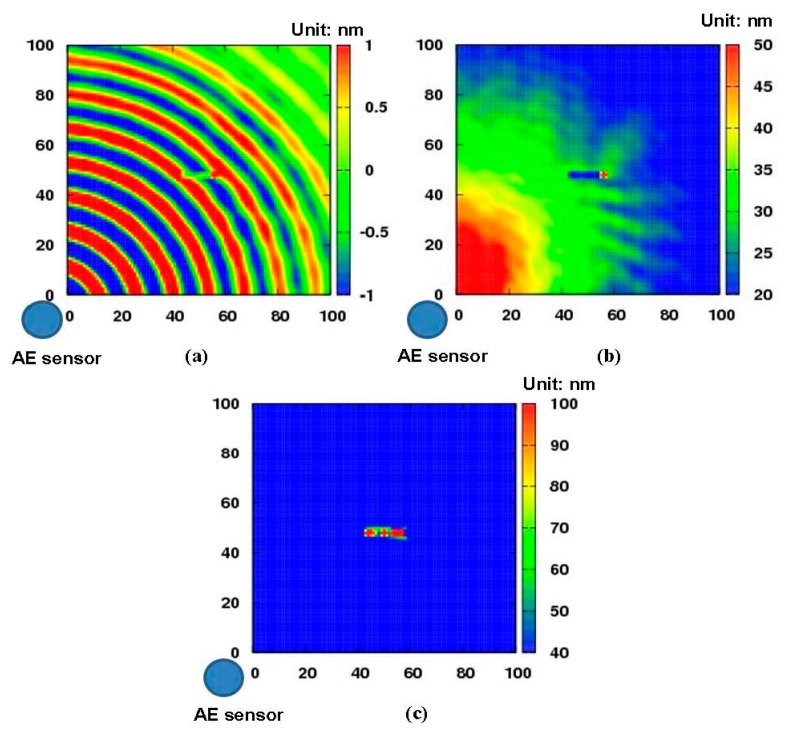
Diagnostic images (a 1 mm depth non-penetrative slit): (**a**) snapshot at 100 μs; (**b**) distribution image of kinetic energy; (**c**) the improved diagnostic image.

Figure 7 presents the experimental results of the aluminum plate with a 1 mm depth non-penetrative slit. Figure 7a is the snapshot of Lamb wave propagation in the aluminum plate at the moment of 100 μs, from which the interaction between the Lamb waves and the non-penetrative slit can be identified clearly. Figure 7b shows the result only using Equation (2) as described in Section 2.1, *i.e.*, the distribution image of kinetic energy obtained by summing up the amplitudes of the waveform data of each grid point. There is a suspicious region with red color near the AE sensor position and the health area is marked by gradually changed colors, which might obscure the real damage area. However, compared to Figure 7a, we can see that, basically, the slit can be imaged not only in its position but also in its shape. Therefore, the efficiency of ultrasonic inspection can be improved and overlook of damage can be partially prevented by using Equation (2). Figure 7c demonstrates an improved result of Figure 7b by further using the new algorithm (*i.e.*, Equation (3)). The damage image is clearly distinct from the health area. Therefore, the damage region can be emphasized by using the new algorithm in Section 2.2, which could further prevent the overlook of damage compared to Figure 7b.

### 3.4. Experimental Results for a Non-Penetrative Slit (0.2 mm Depth)

**Figure 8.**
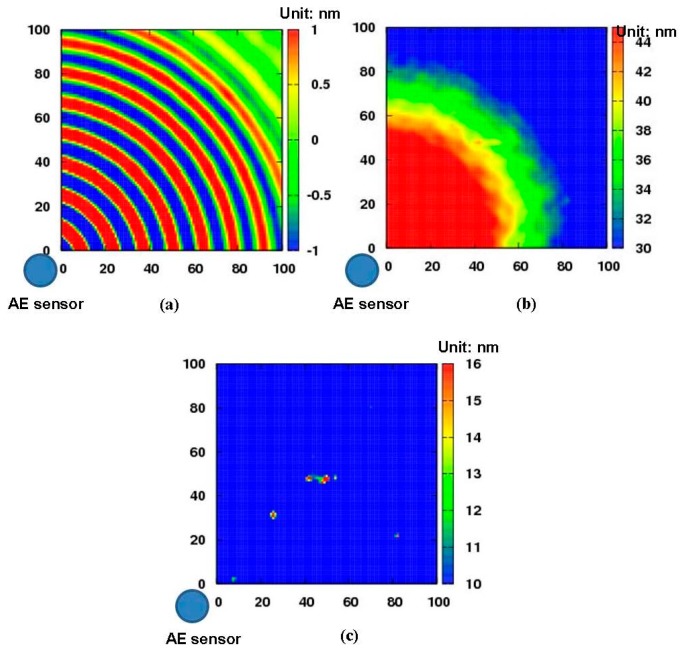
Diagnostic images a 0.2 mm depth non-penetrative slit: (**a**) snapshot at 100 μs; (**b**) distribution image of kinetic energy; (**c**) the improved diagnostic image.

The diagnostic images of the aluminum plate with a 0.2 mm depth non-penetrative slit are shown in Figure 8. Figure 8a is the snapshot of Lamb wave propagation in the aluminum plate at the moment of 100 μs. Compared with Figure 7a, the interaction between the Lamb waves and the slit cannot be clearly identified due to the shallower depth of the non-penetrative slit. The result only using Equation (2) in Section 2.1, is presented in Figure 8b. We can see that it is hard to clearly distinguish the slit area. However, by further using Equation (3) in Section 2.2, the shallow non-penetrative slit can be imaged clearly as shown in Figure 8c. Thus, the new algorithm is certainly capable of enhancing the quality of the damage diagnostic image, especially for minor defects.

### 3.5. Experimental Results for a Circular Dent

**Figure 9.**
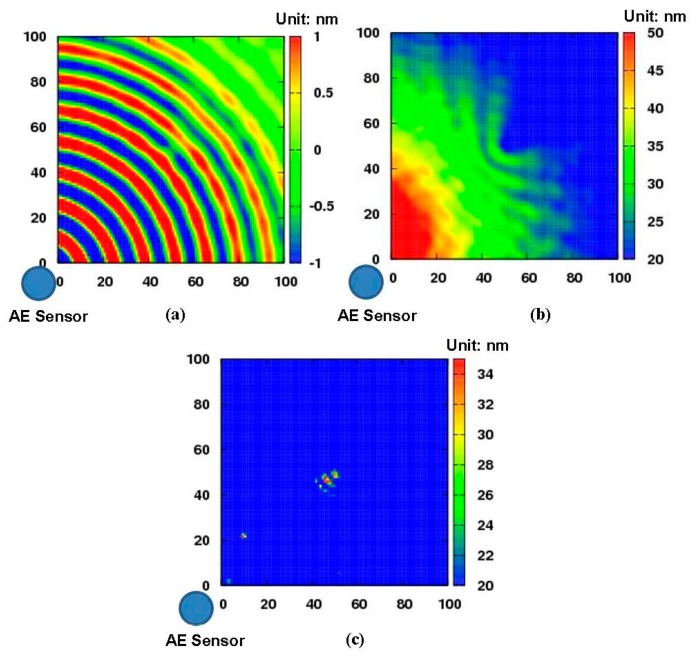
Diagnostic images of a circular dent: (**a**) snapshot at 100 μs; (**b**) distribution image of kinetic energy; (**c**) the improved diagnostic image.

The diagnostic images of the aluminum plate with a 2 mm depth circular dent are presented in Figure 9. Figure 9a is also the snapshot of Lamb wave propagation in the aluminum plate at the moment of 100 μs. We can see that the interaction between the Lamb waves and the circular dent is weak and indistinct and the circular dent cannot be inspected. The result obtained by only using Equation (2) in Section 2.1 is shown in Figure 9b, from which the position of a defect can be possibly identified. However, it is difficult to identify its shape and size. By further applying the new signal processing algorithm using Equation (3), the improved diagnostic image presented in Figure 9c is obtained. Compared with Figure 9b, it can be seen that the position and area of the circular dent can be approximately sketched in Figure 9c. From our previous experiences in [17], it can be expected that by employing multiple AE sensors placed around the inspection region, the overall area and shape of the circular dent damage can be imaged more clearly.

## 4. Conclusions

To construct the distribution images of the kinetic energy of particles in inspected structures, based on the Moore neighbor tracing method, we have put forward a new signal processing algorithm to improve the reliability of the Lamb wave visualization technique. The new algorithm employs the characteristic that the kinetic energy of a particle located near or in a defect would suddenly change when elastic waves encounter the damage during the wave propagation process. To confirm the effectiveness of the new algorithm, three aluminum plates with two non-penetrative slits of different depths and a circular dent, respectively, at the center of the plate surface, were used in experiments. Using the wave signals collected in the inspection region, the snapshot of wave propagation at 100 μs, the distribution image of kinetic energy and the improved diagnostic image obtained by using the new algorithm are illustrated. From those results, we can see that the improved diagnostic image can emphasize the images of all the three damages more clearly compared with the other two kinds of results, which proves the effectiveness of the new algorithm.
